# Development of muscular dystrophy in a CRISPR-engineered mutant rabbit model with frame-disrupting *ANO5* mutations

**DOI:** 10.1038/s41419-018-0674-y

**Published:** 2018-05-22

**Authors:** Tingting Sui, Li Xu, Yeh Siang Lau, Di Liu, Tingjun Liu, Yandi Gao, Liangxue Lai, Renzhi Han, Zhanjun Li

**Affiliations:** 1 Jilin Provincial Key Laboratory of Animal Embryo Engineering, Institute of Zoonosis, Jilin University, 130062 Changchun, China; 2 Department of Surgery, Davis Heart and Lung Research Institute, Biomedical Sciences Graduate Program, Biophysics Graduate Program, The Ohio State University Wexner Medical Center, Columbus, OH 43210 United States

## Abstract

Limb girdle muscular dystrophy type 2L (LGMD2L) and Miyoshi myopathy type 3 (MMD3) are autosomal recessive muscular dystrophy caused by mutations in the gene encoding anoctamin-5 (*ANO5*), which belongs to the anoctamin protein family. Two independent lines of mice with complete disruption of *ANO5* transcripts did not exhibit overt muscular dystrophy phenotypes; instead, one of these mice was observed to present with some abnormality in sperm motility. In contrast, a third line of *ANO5*-knockout (KO) mice with residual expression of truncated *ANO5* expression was reported to display defective membrane repair and very mild muscle pathology. Many of the *ANO5*-related patients carry point mutations or small insertions/deletions (indels) in the *ANO5* gene. To more closely mimic the human *ANO5* mutations, we engineered mutant *ANO5* rabbits via co-injection of Cas9 mRNA and sgRNA into the zygotes. CRISPR-mediated small indels in the exon 12 and/or 13 in the mutant rabbits lead to the development of typical signs of muscular dystrophy with increased serum creatine kinase (CK), muscle necrosis, regeneration, fatty replacement and fibrosis. This novel *ANO5* mutant rabbit model would be useful in studying the disease pathogenesis and therapeutic treatments for *ANO5*-deficient muscular dystrophy.

## Introduction

The limb girdle muscular dystrophies (LGMDs) are a diverse group of childhood and adult onset muscle diseases, characterized by progressive weakness of the hip and shoulder girdles and of the lower limbs, with muscle atrophy. The prevalence of LGMDs is about between 1 in 14,500 and 1 in 45,000 in the world^1,2^. To date, several genes have been identified in LGMDs including anoctamin-5 (*ANO5*), the causative gene for LGMD2L.

The *ANO5* gene encodes *ANO5*, a member of the anoctamin/TMEM16 family of proteins with putative calcium-activated chloride channel and/or phospholipid scramblase activities^3^. The dominant mutations of *ANO5* have been linked to gnathodiaphyseal dysplasia (GDD)^4^, while recessively inherited mutations cause LGMD2L and Miyoshi myopathy type 3 (MMD3)^5–7^. The phenotypical presentation of patients with *ANO5* mutations varies remarkably, but symptoms typically begin in adulthood (age 20–50) with proximal lower limb weakness, high serum creatine kinase (CK) levels, asymmetric muscle atrophy and weakness, and sarcolemmal lesions, similar to dysferlinopathy^8,9^.

The molecular and cellular functions of *ANO5* are not well understood. Due to the significant sequence homology with other anoctamin proteins, it is believed that *ANO5* may function as either a calcium-activated chloride channel, or a phospholipid scramblase or both^10,11^. Similar to other anoctamin proteins, *ANO5* is predicted to have 10 membrane-spanning helices and a hydrophilic cavity exposed to the lipid bilayer that likely represents the site of catalysis for phospholipid scrambling^12^. *ANO5* is found in skeletal muscle, cardiac muscle, bone, testis and thyroid^13^, where it is primarily localized on the endoplasmic reticulum (ER) membrane^14^. Due to the phenotypical similarity between anoctaminopathy and dysferlinopathy, it is suggested that *ANO5* may be involved in the membrane repair process. Moreover, the membrane repair efficiency was found to be defective in fibroblasts from a patient with *ANO5* mutation and in skeletal muscle cells from an *ANO5*-knockout (KO) mouse model^15^. However, it remains to be determined how the ER-localized *ANO5* plays a role in the cell plasma membrane repair.

At present, several *ANO5*-KO mice have been reported with different pathological outcomes^15^. In particular, two groups generated the *ANO5*-KO mice by disrupting the first or second exon of the gene, and the *ANO5* transcripts were reported to be completely disrupted. These two *ANO5*-KO mice did not present with an overt myopathy. However, an *ANO5* gene trapped mouse model with the insertion in intron 8 showed residual expression of mutant *ANO5* transcript and presented with a mild muscular dystrophy phenotype. It is not very clear what exactly underlies the pathological variations in these *ANO5*-KO mice, however, the nature and localization of the mutations and the species difference are most likely responsible for the poor modeling of *ANO5*-deficient muscular dystrophy in mice. Of note, rabbits are considered better animal models than mice in recapitulating some human diseases because of the higher similarity in terms of physiology, anatomy and genetics with human beings than mice^16^.

Here we report the generation of *ANO5* mutant rabbits, which recapitulate many features of LGMD2L patients by cytoplasmic microinjection of Cas9 mRNA and single guide RNA (sgRNA). We showed that engineered small insertions/deletions (indels) in the *ANO5* gene of rabbits lead to muscle degeneration/regeneration, atrophy and elevation of serum CK levels. This novel *ANO5*-KO rabbit model could be used for pathogenesis studies and therapeutic development for *ANO5*-deficient muscular dystrophy.

## Result

### Design of CRISPR/Cas9 system and generation of *ANO5-*KO rabbits

**Fig. 1:**
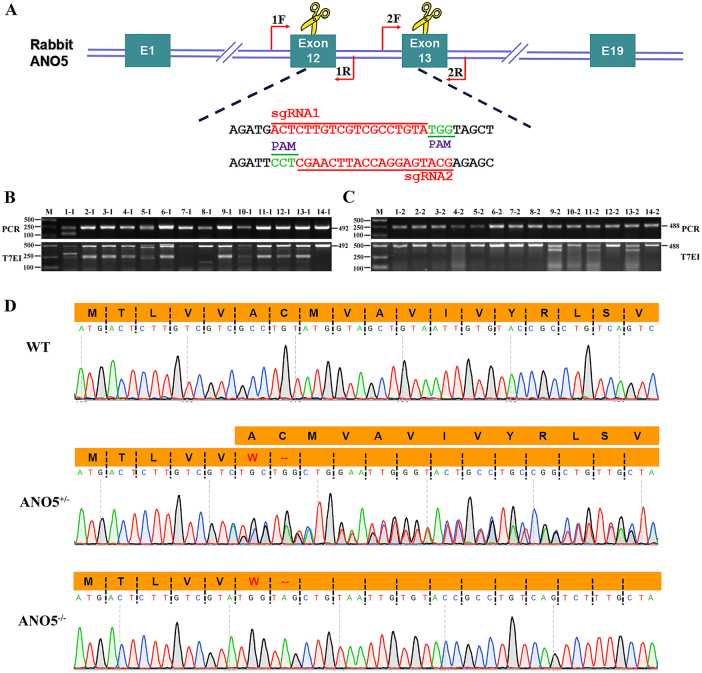
Generation of *ANO5* mutant rabbit. **a** Schematic diagram shows the two different gRNAs targeting exon 12 and exon 13, respectively. **b**, **c** PCR analysis showing different mutations by the T7E1 assay using two different set of primers targeting exon 12 and exon 13, respectively. Gel images have been cropped. M, DL2000, has been used to indicate band size. Black line indicates the WT allele (492 and 488 bp). **d** Sanger sequencing results of the *ANO5* RT-PCR product and the corresponding changes of amino acid sequences at the target site. Nonsense mutation was detected in puppies #7. ‘--’ indicates stop codon sites

To generate an *ANO5-KO* model, we designed a pair of sgRNAs targeting exon 12 and exon 13 to disrupt the open reading frame (ORF) of *ANO5* in rabbit (Fig. 1a). To test the efficiency of gene targeting of *ANO5* in zygotes, the mixed Cas9 mRNA and sgRNAs were microinjected into the zygotes, and cultured until the blastocyst stage. As shown in Table 1, 78.6% of injected embryos (*N* = 152) developed into the blastocyst stage, among which 75.4% carried mutations in *ANO5* at the target sites. There were no significant differences in the developmental rate between the non-injected embryos and microinjected embryos (*p* > 0.05). These results showed that the dual sgRNA-directed CRISPR/Cas9 system is efficient for generation of mutations in the *ANO5* gene in rabbit embryos.

**Table 1:** Summary of embryo microinjections of Cas9/sgRNA in zygotes

	Replications	No. zygotes	Two-cell (%)	Morula (%)	Blastocyst (%)	Blastocyst with mutation (%)
Non-injection	4	150	95.7 ± 0.92	82.1 ± 1.21	78.58 ± 0.78	
Injection	4	152	94.7 ± 0.34	83.9 ± 1.63	77.4 ± 1.82	75.4 ±1.59*

Data are presented as mean ± SEM, and analyzed by *t-*tests using Graphpad Prism software 7.0.

*p<0.05 compared vs blastocyst

To generate *ANO5-*KO rabbit, the 128 injected zygotes were transferred into the oviducts of four surrogate rabbits. Three of four surrogates were pregnant to term and produce 26 live pups (Table 2). The ear tissue of each animal was collected for genotyping. The mutations type was determined by the T7E1 assay and Sanger sequencing of the PCR products. As shown in Fig. 1b, c and Fig. S1, 13 of 26 (50%) newborn pups carried *ANO5* mutations. We chose the rabbits with 7-bp deletion for further analysis. Sanger sequencing of the *ANO5* RT-PCR product covering the exon 12 showed that the mutant transcript was expressed with a predicted premature stop codon at the deletion site (Fig. 1d). In order to examine the off-target effects in these *ANO5-*KO rabbits, the PCR products of top 12 potential off-target sites were subjected to Sanger sequencing and the T7E1 assay. No off-target mutations were detected at these potential sites in the *ANO5*-KO rabbits (Fig. S2).

**Table 2:** Generation of the *ANO5*-KO rabbits using CRISPR/Cas9 system

Recipients	Embryos transferred	Pregnancy	Pups obtained (% transferred)	Pups with mutations (% pups)
1	31	Yes	8 (25.8%)	4 (50.0%)
2	28	Yes	5 (17.9%)	1 (20.0%)
3	32	Yes	13 (40.6%)	8 (61.5%)
4	33	No	0 (0%)	0 (0%)

### Phenotype characterization of *ANO5-*KO rabbits

**Fig. 2:**
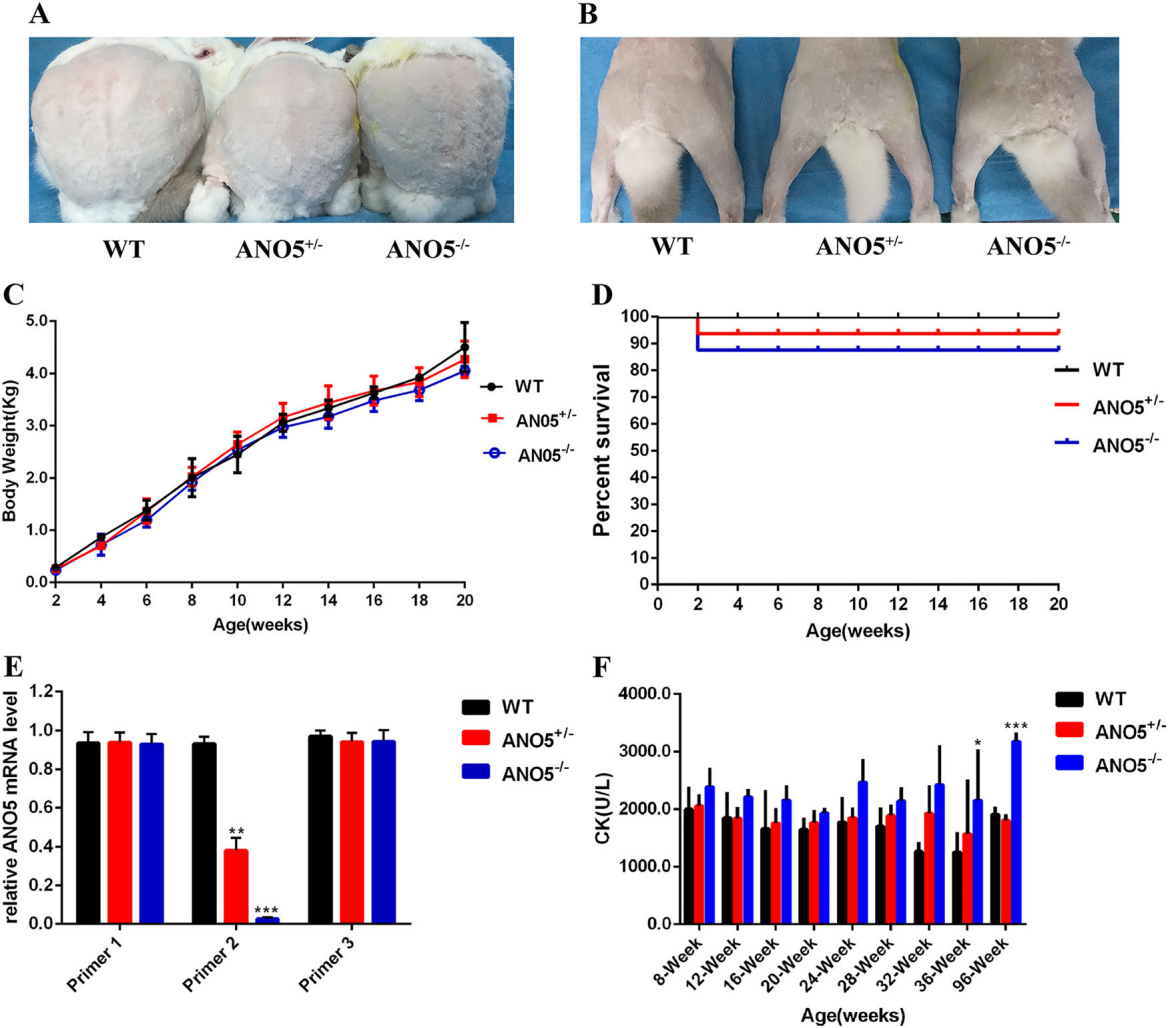
Characterization of *ANO5* mutant rabbit. **a**, **b** The representative rabbit hind-limbs show no significant different among WT, *ANO5*^+/-^ and *ANO5*^-/-^ rabbit muscles at 8 weeks (*n* = 3). **c** Body weight, **d** percent survival rate, **e** *ANO5* mRNA expression level by three different set of primers targeting upstream or downstream region of *ANO5* away from the gRNA target sites and exon 12 region, and **f** serum CK levels of WT, *ANO5*^+/-^ and *ANO5*^-/-^ rabbit (*n* = 6). *p<0.05; **p<0.01;***p<0.001

Due to the unavailability of a good anti-*ANO5* antibody to detect rabbit *ANO5* protein, we used quantitative reverse transcriptase-PCR (RT-PCR) to determine the effect of the engineered mutations in *ANO5* on its expression in the KO rabbits using three sets of primers. The total RNA was extracted from gastrocnemius the of KO and WT rabbits. As shown in Fig. 2e, with the primer set2, where the reverse oligo is annealed to the gRNA targeting site in exon 12, the expression of *ANO5* was significantly reduced in *ANO5-*KO rabbits compared with that of the heterozygous or WT controls. However, with the primer set 1 (annealed to the downstream region of *ANO5* away from the gRNA target sites) and 3 (annealed to the upstream region of *ANO5* away from the gRNA target sites), the expression levels of *ANO5* transcripts were found to be similar between the KO and WT rabbits (Fig. 2e). These data suggest that the mutant *ANO5* transcripts were expressed at the similar levels in the *ANO5*-KO rabbits as compared with those of the WT *ANO5* transcripts in WT rabbits.

To determine the gross physiological impact of the engineered *ANO5* mutations, we collected the body weight data biweekly and calculated the mortality rate. As shown in Fig. 2a-c, the *ANO5*-KO rabbits showed no obvious difference as compared with their WT littermates. There were no significant changes in their body weights from 2 to 20 weeks of age compared with the age-matched WT or heterozygous littermates. There were also no significant changes in the mortality of these animals (Fig. 2d).

### Muscular dystrophy presentation in *ANO5*-KO rabbits

**Fig. 3:**
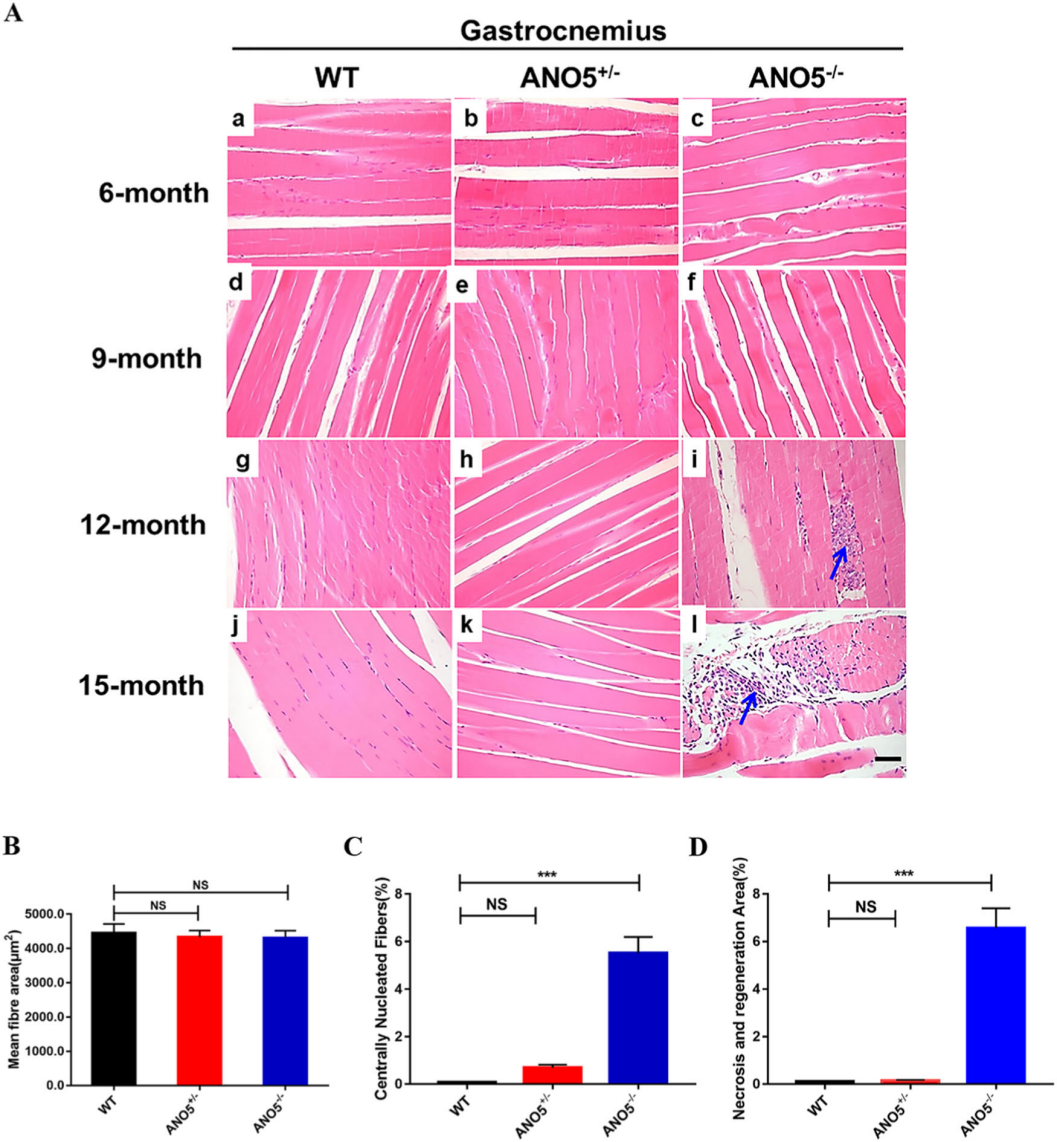
Histological studies in gastrocnemius muscle of *ANO5* mutant rabbit. **a** The histomorphological analysis of WT, *ANO5*^+/-^ and *ANO5*^-/-^ rabbit gastrocnemius muscles by H&E at 6, 9, 12 and 15 months. Arrow indicates the presence of necrosis and regeneration area (H&E). **b** The analysis of muscle fiber area from WT, *ANO5*^+/-^ and *ANO5*^-/-^ rabbit . **c** The analysis of centrally nucleated fibers from WT, *ANO5*^+/-^ and *ANO5*^-/-^ rabbit. **d** The analysis of necrosis and regeneration area from WT, *ANO5*^+/-^ and *ANO5*^-/-^ rabbit. Scale bar: 50 µm. ***p<0.001; *n *= 3

Previous studies have reported that *ANO5* mutant patients had marked elevation of serum CK levels and muscle weakness^8,17^. We evaluated clinical and histopathological features of *ANO5-*KO rabbits. As shown in Fig. 2f, the serum CK was significantly elevated in the *ANO5*-KO rabbits from 9 months of age compared with their age-matched littermate controls. To further examine the histopathology of the *ANO5*-KO rabbits, we performed hematoxylin and eosin (H&E) stainings of the gastrocnemius muscle sections from the rabbits at 6, 9, 12 and 15 months of age and Masson’s trichrome staining of the gastrocnemius at 15 months of age. As shown in Fig. 3a, the *ANO5*^-/-^ rabbits displayed typical muscular dystrophy signs as evidenced by scattered necrotic muscle fibers with inflammatory infiltrates. There was no statistical difference in the average muscle fiber size between the wild-type and *ANO5*^-/-^ rabbits (Fig. 3b). However, *ANO5* rabbits displayed increased percentage of centrally nucleated fibers and necrotic area (Fig. 3c, d). The appearance of these phenotypes was typically observed starting at 12 months of age.

**Fig. 4:**
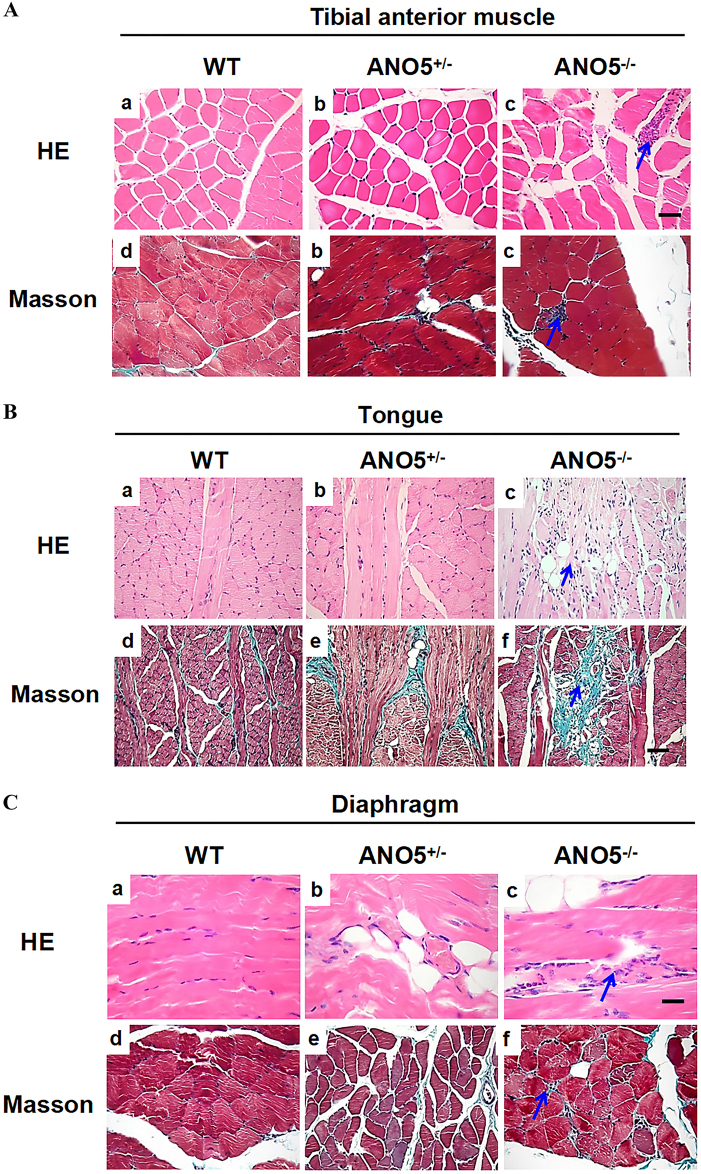
Histological examination of tibialis anterior, diaphragm and tongue of *ANO5* mutant rabbit. **a** The histomorphological characteristic of WT and *ANO5*^-/-^ rabbit tibialis anterior muscles by H&E and Masson, at 15 months. **b** The histomorphological characteristic of WT and *ANO5*^-/-^ rabbit tongue muscles by H&E and Masson, at 15 months. **c** The H&E staining shows the presence of muscle degeneration and irregular muscle fiber size in mutant rabbit diaphragm, whereas the Masson staining showed the collagen-rich tissue in fibrotic region (*n* = 3). Scale bar: 50 µm

Similarly, muscle necrosis was also observed in *tibialis anterior* muscle (Fig. 4a). Again, the fiber size in *tibialis anterior* muscle was not significantly different compared with their control counterparts (Fig. S4). Moreover, the pathological alterations were mostly evident in the tongue muscle (Fig. 4b), which showed extensive muscle degeneration, fibrosis and fatty replacement.

**Fig. 5:**
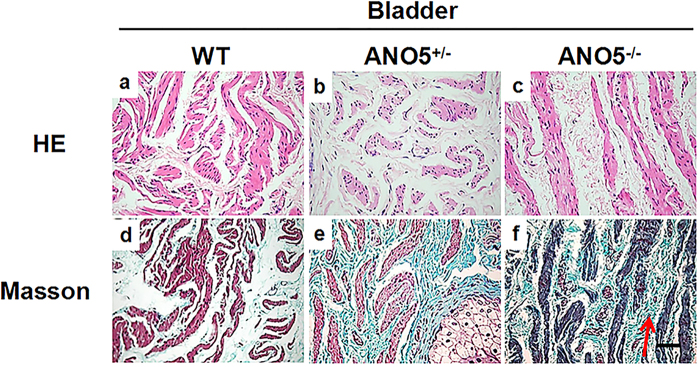
The H&E and Masson’s staining of bladder tissues from WT and *ANO5*^*-/-*^ rabbits (*n* = 3). **a-c** Representative images of H&E staining from WT, *ANO5*^+/-^  and *ANO5*^-/-^rabbit. **d-f** Representative images of Masson's staining from WT, *ANO5*^+/-^ and *ANO5*^-/-^rabbit. Scale bar: 50 µm

The diaphragm muscles were also found to have extensive muscle degeneration and fibrosis (Fig. 4c), whereas the average fiber area was no significantly changed (Fig. S5). Interestingly, the pathological changes was also noted in smooth muscle of *ANO5*^-/-^ rabbits. As shown in Fig. 5, the bladder of the *ANO5*^-/-^ rabbits displayed extensive fibrosis. Therefore, these studies suggest that the loss-of-function mutations in the *ANO5* gene lead to the pathological changes in both skeletal and smooth muscles.

**Fig. 6:**
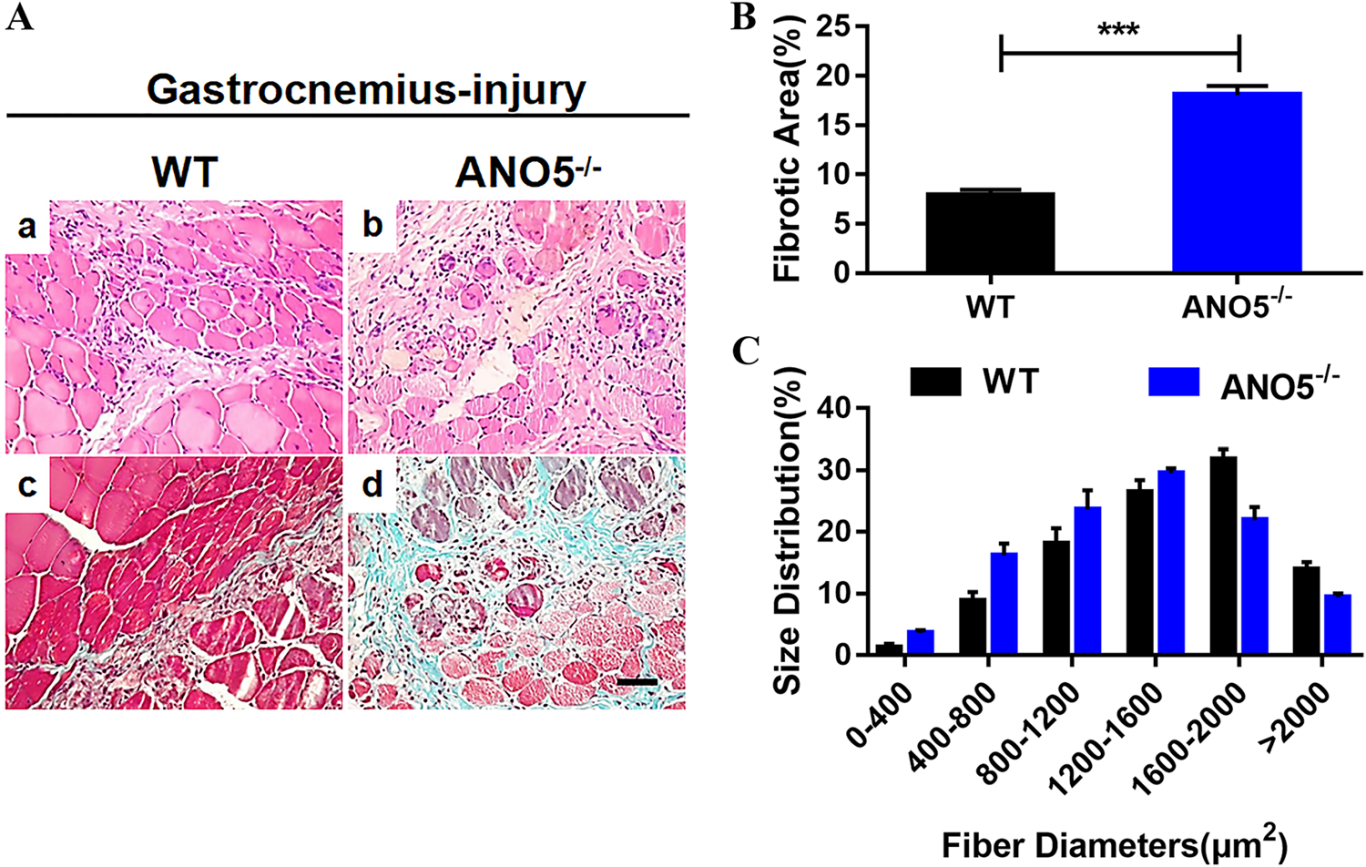
Cardiotoxin-induced muscle injury and regeneration. **a** H&E (top) and Masson’s trichrome (bottom) stained muscle sections of WT and *ANO5*^-/-^ rabbits at 14 days post-cardiotoxin injection. **b** Quantification of relative fibrotic area in WT and *ANO5*^-/-^ rabbit muscles at 14 days after cardiotoxin injection. **c** Fiber size distribution of WT and *ANO5*^-/-^ *gastrocnemius* muscles at 14 days post-cardiotoxin injection. Scale bar: 50 µm. ***p < 0.001; *n* = 3

Finally, we studied whether *ANO5* plays a role in muscle regeneration following cardiotoxin-induced injury in rabbits. At 14 days after cardiotoxin injection, the *gastrocnemius* muscle from the wild-type rabbits showed typical signs of regeneration as evidenced by the presence of central nuclei (Fig. 6a). However, the injured *ANO5*^*-/-*^ muscles showed dramatic increase in fibrosis (Fig. 6a, b) and delayed regeneration with increased number of smaller muscle fibers (Fig. 6c). These results suggest that *ANO5* plays a role in muscle regeneration.

## Discussion

In the present study, we generated a novel rabbit model with *ANO5* mutations via the zygote injection of Cas9 mRNA and a pair of sgRNA targeting the rabbit *ANO5* gene. Our data demonstrate that CRISPR-induced indels within the exon 12 or 13 of the *ANO5* gene lead to the development of pathological alterations in various muscles of the rabbit, resembling human patients with *ANO5* mutations. Three independent laboratories including our own group reported the phenotypic results of the *ANO5*-KO mice, which were generated by traditional gene KO or gene-trapping strategy^15,18,19^. Two of these *ANO5*-KO mice, produced by disruption of either exon 1^18^ or exon 2^19^, do not exhibit any obvious muscle pathology; whereas the third line of *ANO5*-KO mice, produced by gene-trapping between exons 8 and 9^15^, showed very mild myopathy as evidenced by a very small increase in serum CK and central nucleation. In contrast, the rabbits carrying small indels in exons 12 or 13 generated in the present study showed typical dystrophic features including muscle necrosis, regeneration, fatty replacement and fibrosis. Several possibilities may explain the different outcomes of muscle pathology in these *ANO5* mutant models.

First, the species and genetic background may play an important role in muscle pathology associated with *ANO5* mutations. There are many examples in the literature that the severity of disease could be affected by genetic backgrounds and/or species. For example, *mdx* mice, a widely used mouse model of Duchenne muscular dystrophy (DMD), have much milder pathology as compared with DMD patients^20^; the severity of muscular dystrophy in dysferlin-null mice depends on their genetic background^21^; a common disease-associated missense mutation in α-sarcoglycan fails to cause muscular dystrophy in mice^22^. Studies have shown that genetic modifiers may affect the disease outcomes in DMD and dysferlinopathy^23,24^. Thus, this raises a possibility that genetic modifiers may also contribute to the disease manifestation in muscular dystrophy associated with *ANO5* mutations. In support of this, it has been shown that there is a wide variation of pathological outcomes among patients even with the same or similar *ANO5* mutations^25,26^. Future investigations would be required to ascertain the involvement of genetic modifiers in the disease progression associated with *ANO5* mutations and identify them. Elucidation of the genetic modifiers affecting muscular dystrophy associated with *ANO5* mutations would not only shed light into the pathogenesis but may also offer us the opportunity to identify novel therapeutic targets to treat the disease.

Second, it is also plausible that the nature of different mutations is responsible for the different outcomes in these animal models. The complete disruption of *ANO5* expression in the two lines of *ANO5*-KO mice does not cause muscle pathology, indicating that mice may have an efficient compensatory mechanism to maintain muscle function in the complete absence of *ANO5*^19^. There are a total of 10 members of anoctamin protein family and some overlapping expression in skeletal muscle^27^. It is possible that these anoctamin homologs may compensate for the complete loss of *ANO5* in mice. On the other hand, intragenic frame-terminating or shifting mutations in either the gene trapped mice or our *ANO5* mutant rabbits result in muscle disease. Recently, Nigro and his colleagues screened a cohort of 786 undiagnosed patients with LGMD or nonspecific myopathic features using next-generation sequencing, and found that 33 out of 786 patients carry *ANO5* mutations in both allele (either homozygous or compound heterozygous). Interestingly, the majority of these 33 patients carry at least one allele with a premature termination mutation (either nonsense or frame-shifting; only 9 carry both missense mutations)^28^. A common theme with the gene-trapping mouse, our CRISPR rabbit and human patients lies in that truncated *ANO5* expression is expected, although the lack of a good antibody with high affinity and specificity to detect mouse or rabbit *ANO5* makes it challenge to draw a firm conclusion. We recently showed that a LGMD2L patient carrying a frame-shifting mutation in *ANO5* had truncated *ANO5* expression, which is also prone to form intracellular aggregates, highlighting its potential contribution to the pathogenesis of muscular dystrophy. Thus, it appears that expression of truncated *ANO5* peptides may have more deleterious effects on muscle health and function as compared with the complete absence of *ANO5* protein, potentially via inhibiting the compensatory functions of other anoctamin proteins. Future studies with CRISPR gene-editing technology would allow us to unveil the compensatory mechanisms, as well as the mechanisms by which truncated *ANO5* inhibits such compensatory pathways.

Taken together, the *ANO5* mutant rabbit model generated by CRISPR gene-editing recapitulates many aspects of muscular dystrophy associated with *ANO5* mutations in human patients. This new model would facilitate the basic research to understand the pathogenesis of *ANO5*-associated muscular dystrophy and the physiological function of *ANO5*, and the translational studies to develop novel therapeutic strategies for the treatment of this disease.

## Materials and methods

### Animals and ethics statement

The New Zealand rabbits used in this study were maintained at the Laboratory Animal Center of Jilin University. All rabbit experiments in this study were reviewed and approved by the Animal Care and Use Committee of Jilin University.

### CRISPR/Cas9 sgRNA preparation, embryo microinjection and embryo transfer

The CRISPR/Cas9 sgRNA was designed and assembled as previously described^29^. The annealed sgRNA oligos were cloned into the *Bbs*I sites of pUC57-T7-sgRNA cloning vector (Addgene ID 51306). The vector of pUC57-T7-sgRNA was PCR amplified using the T7 primers (T7-F: 5′-GAAATTAATACGACTCACTATA-3′ and T7-R: 5′-AAAAAAAGCACCGACTCGGTGCCAC-3′), and the PCR products were transcribed in vitro with MAXIscript T7 Kit (Ambion) and purified by miRNeasy Mini Kit (Qiagen) according to the manufacturer's instruction.

The 3xFLAG-NLS-SpCas9-NLS vector (Addgene ID 48137) was linearized with *Not*I and transcribed in vitro using the mMessage mMachine SP6 Kit (Ambion) and the RNeasy Mini Kit (Qiagen) according to the manufacturer's instruction. The microinjection procedure and embryo transfer was essentially the same as we previously described^30^.

### Mutation detection in pups by PCR and sequencing

The genomic DNA from *ANO5-*KO and WT rabbits were extracted from a small piece of ear tissue using the TIANamp Genomic DNA Kit (TIANGEN, Beijing, China) according to the manufacturer’s instruction. The sgRNA target sites were amplified by PCR using the primers (forward 1, 5′-CCCATATGCCTTGTTCTATT-3′; reverse 1, 5′-GCATGATTAGGAACCCTTT-3′; forward 2, 5′-CCCTCTGACTCACAAATAAA-3′; reverse 2, 5′-TCATAGCTTACCACCAAATC-3′). The PCR products were gel purified and cloned into pGM-T vector (Tiangen, Beijing, China). A minimum of 14 positive clones were sequenced and analyzed using DNAman.

### T7EI cleavage assay

The T7EI cleavage assay was performed as described previously^31^. Briefly, the PCR products were purified, denatured and then re-annealed in NEBuffer 2 (NEB) using a thermocycler. Hybridized PCR products were digested with T7 endonuclease 1 (NEB, M0302L) for 30 min at 37 °C and subjected to 2% agarose gel electrophoresis.

### Off-target analysis

The potential off-target sites were predicted by online CRISPR Design tool developed by Zhang’s group at MIT (http://crispr.mit.edu/). The PCR products for the top 12 off-target sites using the primers listed in Supplementary Table S1 were subjected to the T7EI assay and Sanger sequencing.

### RNA isolation, RT-PCR and qRT-PCR

The total RNA was isolated from gastrocnemius muscle of WT and *ANO5*-KO rabbits using TRNzol-A+ reagent (Tiangen, Beijing, China), and treated with DNase I (Fermentas). The first-strand complementary DNA (cDNA) was synthesized using the cDNA first-strand synthesis kit (Tiangen, Beijing, China). The cDNA was used for RT-PCR and quantitative RT-PCR (qRT-PCR) analyses to examine the expression of *ANO5*. The primers used for RT-PCR and qRT-PCR were shown in Table S2. The qRT-PCR was performed using the BioEasy SYBR Green I Real Time PCR Kit (Bioer Technology, Hangzhou, China), and the 2-ΔΔCT formula was used to analyze the gene expression, *Gapdh* was used as a reference gene. All experiments were repeated three times. The data were expressed as the mean ± S.E.M.

### Body weight, survival and statistical analysis

The body weight of age- and gender-matched WT and *ANO5-*KO rabbits were measure biweekly. All data were expressed as the mean ± S.E.M., and a minimum of three individual animals of each genotype were used in all experiments. The data were analyzed by the Student’s *t*-test using Graphpad Prism 7.0 software. A probability of *p* *<* *0.05* was considered statistically significant.

### Serum biochemistry analysis

The blood samples were collected into heparinized tubes from the ear vein, and sera were prepared by precipitation and centrifugation. The serum CK was measured using the CK Test Kit (*N*-acetyl-l-cysteine method).

### Histology analysis

Various tissues including *gastrocnemius*, *tibial anterior*, *tongue*, *diaphragm* and *bladder* were collected from *ANO5*-KO and WT rabbits (euthanized at 6, 9, 12 and 15 months of age). The tissues were fixed in 4% paraformaldehyde at 4 °C, dehydrated in increasing concentrations of ethanol (70% for 6 h, 80% for 1 h, 96% for 1 h and 100% for 3 h), cleared in xylene and embedded in paraffin for the histological examination. The 5-μm sections were cut for H&E^32^ and Masson’s trichrome^33^ as previously described. The stained sections were imaged with a Nikon TS100 microscope.

### Morphometric analysis of myofibers

The H&E-stained cross-sections of *gastrocnemius* and *tibial anterior* muscles from the *ANO5*-KO and WT rabbits at the age of 15 months were analyzed for fiber size. A minimum of three different regions were counted per section. The fiber size was calculated using ImageProPlus 6.0 software (Media Cybernetics, Silver Spring, MD, USA).

### Cardiotoxin-induced muscle injury and regeneration

To induced muscle injury and regeneration, cardiotoxin (diluted to 30 μg/ml with sterile saline, 100 μl) was injected into the left *gastrocnemius* muscles of 12-month-old *ANO5*^*-*/-^ and wild-type rabbits. An equal volume of sterile saline was injected into contralateral muscle as a vehicle control. The muscle biopsies were collected at 14 days post-cardiotoxin injection for histopathology analysis.

## Electronic supplementary material

Figure S1

Figure S2

Figure S3

Figure S4

Figure S5

Table S1

Table S2

Supplementary figure legends
