# Correction: Development of muscular dystrophy in a CRISPR-engineered mutant rabbit model with frame-disrupting *ANO5* mutations

**DOI:** 10.1038/s41419-020-2659-x

**Published:** 2020-06-17

**Authors:** Tingting Sui, Li Xu, Yeh Siang Lau, Di Liu, Tingjun Liu, Yandi Gao, Liangxue Lai, Renzhi Han, Zhanjun Li

**Affiliations:** 1 Jilin Provincial Key Laboratory of Animal Embryo Engineering, Institute of Zoonosis, Jilin University, 130062 Changchun, China; 2 Department of Surgery, Davis Heart and Lung Research Institute, Biomedical Sciences Graduate Program, Biophysics Graduate Program, The Ohio State University Wexner Medical Center, Columbus, OH 43210 United States

Correction to**:** *Cell Death and Disease*

10.1038/s41419-018-0674-y, published online 22 May 2018

Since publication of this article, the authors noticed that there was an error in the histology images of Fig. 3a. In Fig. 3a, the histology images of the gastrocnemius muscle at 6-months old were mistakenly assembled using the same images at 9-months old. The corrected figure is provided below, and both the PDF and HTML versions of the article have been updated.

**Figure:**
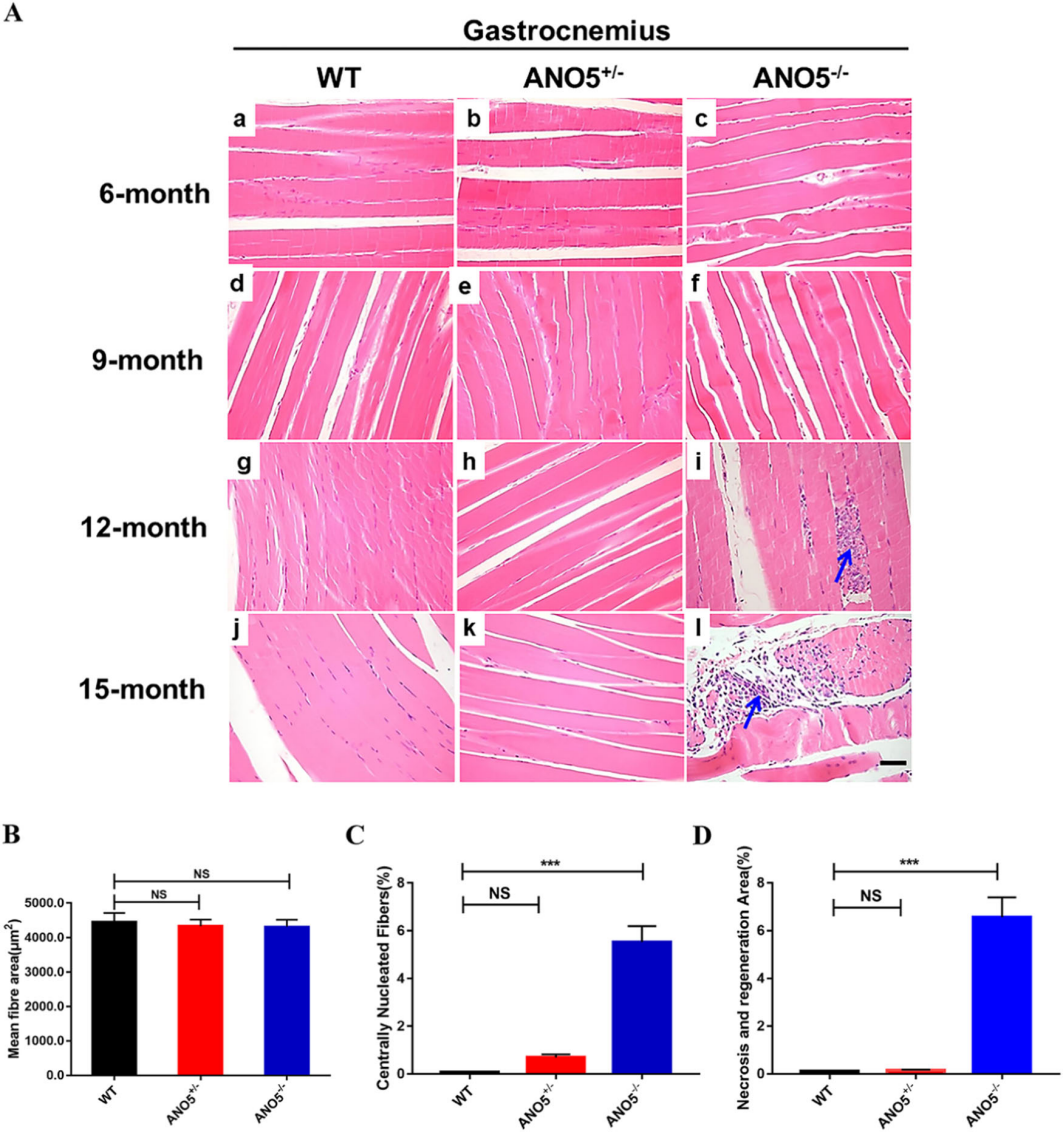
**Fig. 3**

