# Supplementary figures and images for: Development of muscular dystrophy in a CRISPR-engineered mutant rabbit model with frame-disrupting ANO5 mutations
Source: Cell Death Dis. 2018 May 22;9(6):609. doi: 10.1038/s41419-018-0674-y (PMC5964072; doi:10.1038/s41419-018-0674-y)

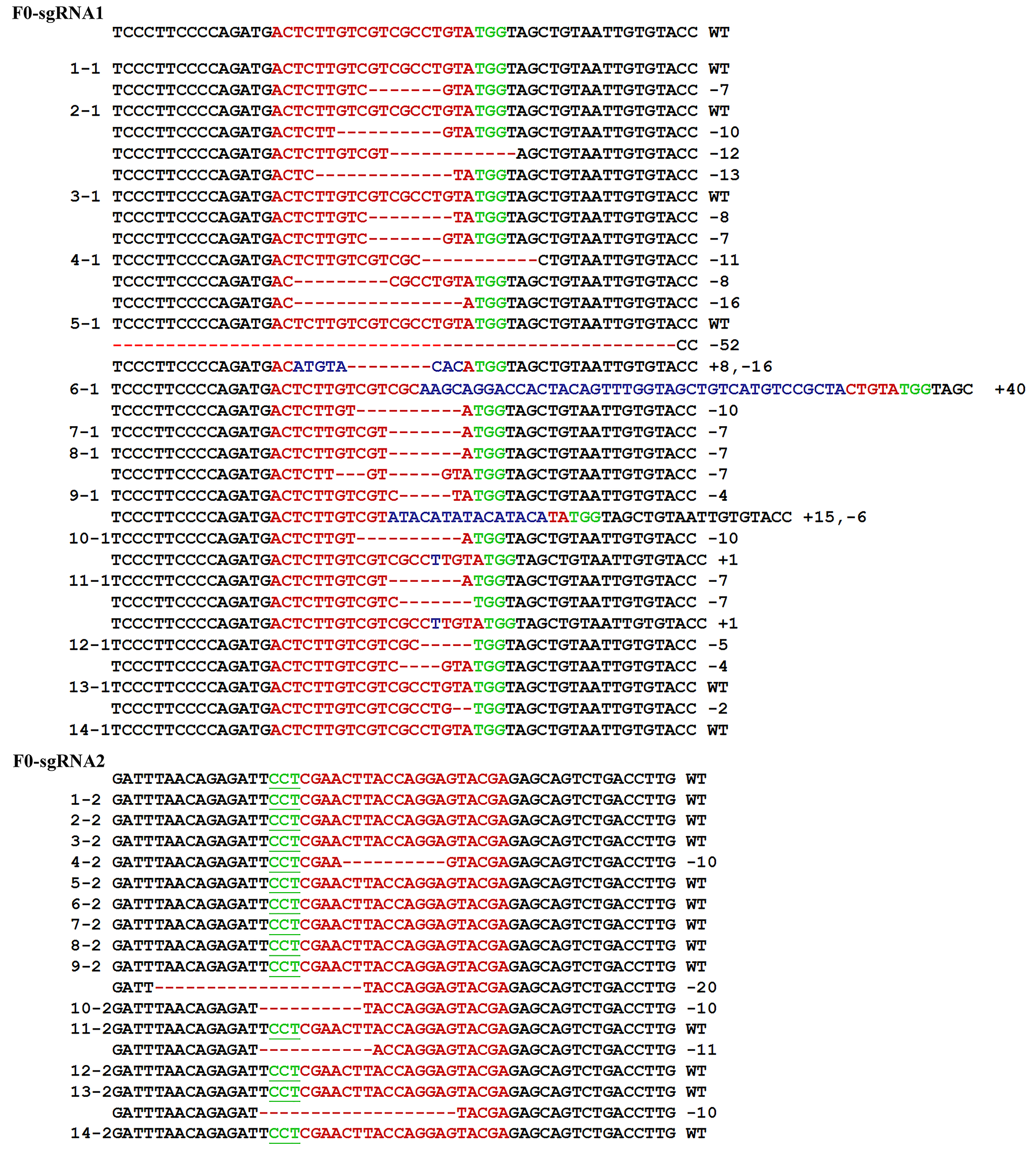

Supplement: Supplementary file 1 — Figure S1 [file 41419_2018_674_MOESM1_ESM.tif]

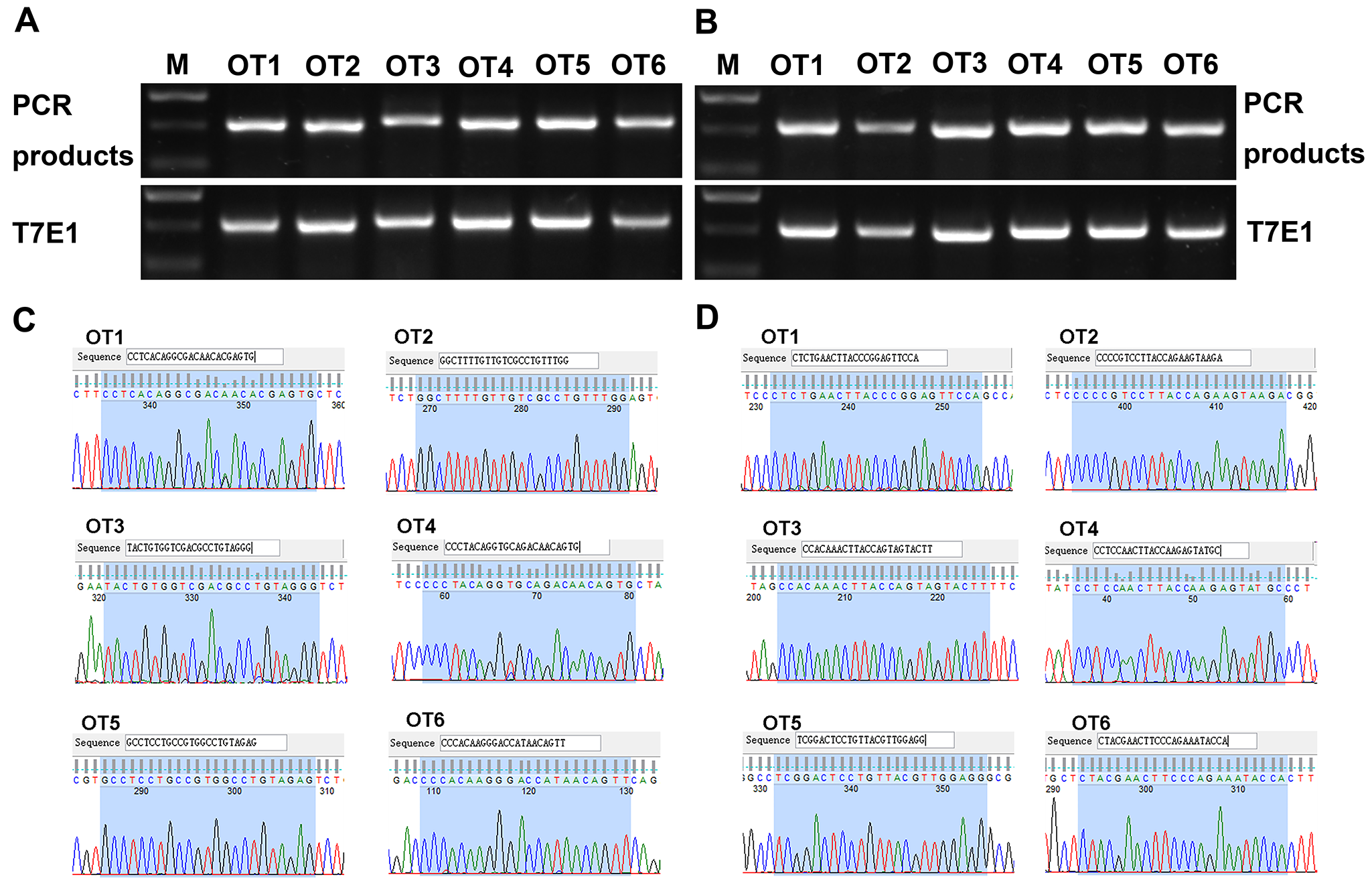

Supplement: Supplementary file 2 — Figure S2 [file 41419_2018_674_MOESM2_ESM.tif]

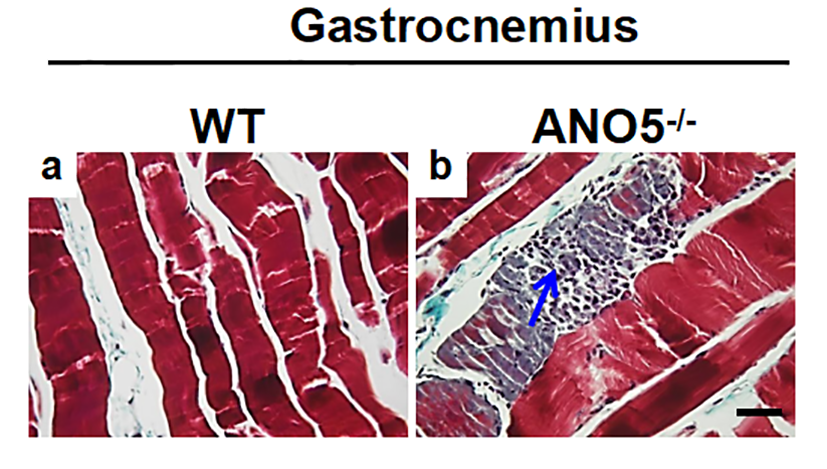

Supplement: Supplementary file 3 — Figure S3 [file 41419_2018_674_MOESM3_ESM.tif]

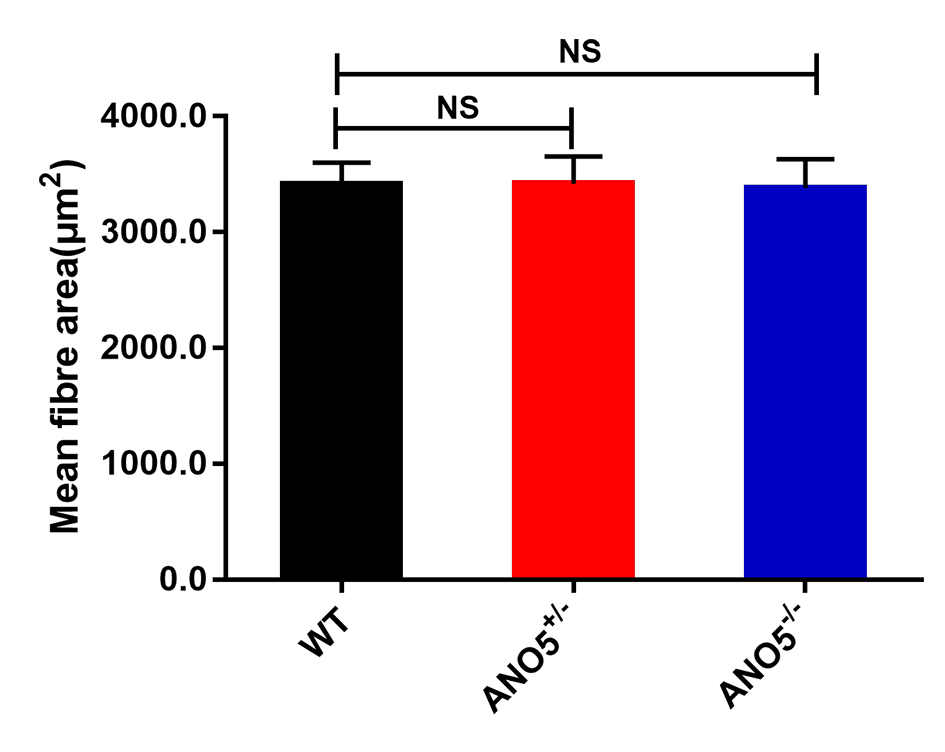

Supplement: Supplementary file 4 — Figure S4 [file 41419_2018_674_MOESM4_ESM.tif]

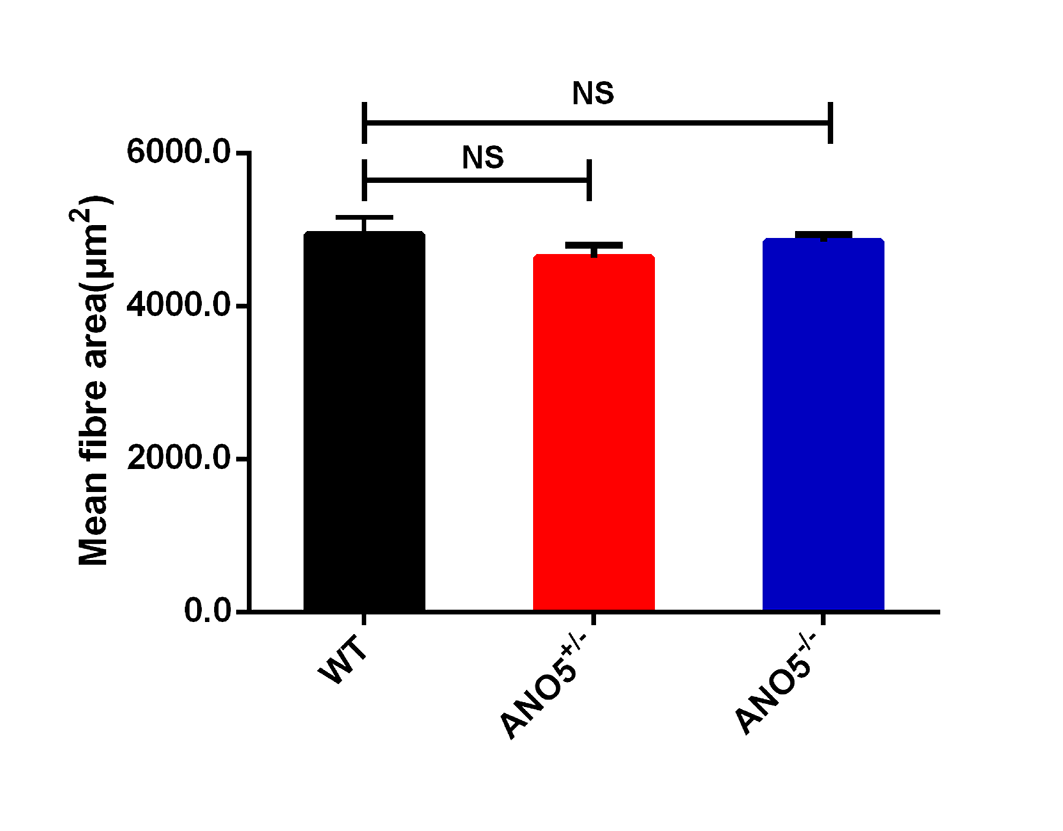

Supplement: Supplementary file 5 — Figure S5 [file 41419_2018_674_MOESM5_ESM.tif]
