# Supplementary material for: Development of muscular dystrophy in a CRISPR-engineered mutant rabbit model with frame-disrupting ANO5 mutations
Source: Cell Death Dis. 2018 May 22;9(6):609. doi: 10.1038/s41419-018-0674-y (PMC5964072; doi:10.1038/s41419-018-0674-y)
Supplement: Supplementary file 6 — Table S1 [file 41419_2018_674_MOESM6_ESM.doc]

Table S1. The primers of potential off-target sites (POTS) used in this study

|  | Potential Off Target Site | Number of mismatch | Position | PCR Primer |
| --- | --- | --- | --- | --- |
| s1 | CACTCGTGTTGTCGCCTGTGAGG | 4 | chrUN0:-1031632 | F：ACATGAACGTACAGCAGGAG  R：CGAGGAGAGCCGTAATGTG |
|  | GGCTTTTGTTGTCGCCTGTTTGG | 4 | chr3:+37778008 | F：GAGTTGACAAGACAGGGAAC  R：GACACCTTAAGACTGCCATC |
|  | TACTGTGGTCGACGCCTGTAGGG | 4 | chr15:+17064254 | F：GCTGGTGGTAGAGATGTTAAG  R：GAGTTACTGAATAGTGGGAGTG |
|  | CACTGTTGTCTGCGCCTGTAGGG | 4 | chrUN0:+335002 | F：CCCTGAGAAACATCCACTATG  R：CATGCATCTGAGACCTTCTATT |
|  | GCCTCCTGCCGTGGCCTGTAGAG | 4 | chr7:+59874 | F：CAGGCTGACGTAGCATTAC  R：GGATCCATTCACCCAGAAAT |
|  | AACTGTTGTGGTCCCCTGTAGGG | 4 | chr9:-13736388 | F：GTCTGTTTCTTCTGGCTGAG  R：CCTTGGTCTTTCCCATCAC |
| **s2** | TGGAACTCCGGGTAAGTTCAGAG | 4 | chrUN0:-905782 | F：GGTTGGAAATGGATGAACTTG  R：GGATGAACAGGTCGTTGAA |
|  | TCTTACTTCTGGTAAGGACGGGG | 4 | chr9:-115934571 | F：TTCTTCTGTCTCCCTGATGA  R：GAGAGGCGTTTCCGAATAAA |
|  | AAGTACTACTGGTAAGTTTGTGG | 4 | chr10:-22335228 | F：CCCTAACGTGTACCCATACTA  R：GTGGTGGGAATGGTGATTT |
|  | TCGGACTCCTGTTACGTTGGAGG | 3 | chrUN0:+656279 | F：GGAGGAGACAGAAACCTCTA  R：GTACATCCAAGAGAGGAGGA |
|  | TGGTATTTCTGGGAAGTTCGTAG | 4 | chr2:-75154379 | F：GCAGATATCGCCTTGGATTA  R：GGCTTTCCTTCTCTGTGAATA |
