# Supplementary material for: Development of muscular dystrophy in a CRISPR-engineered mutant rabbit model with frame-disrupting ANO5 mutations
Source: Cell Death Dis. 2018 May 22;9(6):609. doi: 10.1038/s41419-018-0674-y (PMC5964072; doi:10.1038/s41419-018-0674-y)
Supplement: Supplementary file 7 — Table S2 [file 41419_2018_674_MOESM7_ESM.docx]

Table S2 Primers for qRT-PCR analysis.

| Name | Primers | Sequence | Produce size (bp) | |
| --- | --- | --- | --- | --- |
| Primer1 | Ano5-F1 | CCTGTGGATAAACGAGACTTCA | | 103 |
|  | Ano5-R1 | GGACATGCCAGAATTGCTTATTAT | |  |
| Primer2 | Ano5-F2 | GGAACAGCAACAGCTTCAAC | | 180 |
|  | Ano5-R2 | CATACAGGCGACGACAAGA | |  |
| Primer3 | Ano5-F3 | ATGGGTGACCCGAACCT | | 101 |
|  | Ano5-R3 | CTGCTACTCATGCTCTCTGTTG | |  |
