# Supplementary material for: Development of muscular dystrophy in a CRISPR-engineered mutant rabbit model with frame-disrupting ANO5 mutations
Source: Cell Death Dis. 2018 May 22;9(6):609. doi: 10.1038/s41419-018-0674-y (PMC5964072; doi:10.1038/s41419-018-0674-y)
Supplement: Supplementary file 8 — Supplementary figure legends [file 41419_2018_674_MOESM8_ESM.docx]

**Supplementary figure legends**

**Figure S1:** Mutation detection of the 1-14 F0 rabbits by T-cloning and Sanger sequencing. T-cloning and Sanger sequencing of modified *ANO5* alleles in pups for F0-sgRNA1 and F0-sgRNA2. WT sequence is shown at the top of the targeting sequence. PAM sites are highlighted in green; target sequences are shown in red; deletions (-); insertions are shown in blue; WT, wild-type control.

**Figure S2:** Off-target analysis of *ANO5* KO rabbit. The T7E1 cleavage analysis of five potential off-target sites (POTS) for sgRNA1 (A) and sgRNA2 (B). M, DL2000; OT1-OT6 represented the six POTS for sgRNA1 and OT1-OT6 represented the six POTS for sgRNA2. T-cloning and Sanger sequencing of the six POTS for sgRNA1 (C) and the six POTS for sgRNA2 (D), the region included the 20bp of the POTS and PAM were shown in blue shadow.

**Figure S3:** The Masson staining of gastrocnemius from WT and *ANO5*^-/-^ rabbit. Scale bar: 50 µm.

**Figure S4:** The analysis of tibialis anterior muscle fiber area from WT, *ANO5*^+/-^ and *ANO5*^-/-^ rabbit.

**Figure S5:** The analysis of diaphragm muscle fiber area from WT, *ANO5*^+/-^ and *ANO5*^-/-^ rabbit.
